# Identification of an unauthorized genetically modified bacteria in food enzyme through whole-genome sequencing

**DOI:** 10.1038/s41598-020-63987-5

**Published:** 2020-04-27

**Authors:** Marie-Alice Fraiture, Bert Bogaerts, Raf Winand, Marie Deckers, Nina Papazova, Kevin Vanneste, Sigrid C. J. De Keersmaecker, Nancy H. C. Roosens

**Affiliations:** Sciensano, Transversal activities in Applied Genomics (TAG), J. Wytsmanstraat 14, 1050, Brussels, Belgium

**Keywords:** Biotechnology, Next-generation sequencing, Antimicrobial resistance, Metabolic engineering, Molecular biology

## Abstract

Recently, the unexpected presence of a viable unauthorized genetically modified bacterium in a commercialized food enzyme (protease) product originating from a microbial fermentation process has been notified at the European level (RASFF 2019.3332). This finding was made possible thanks to the use of the next-generation sequencing technology, as reported in this study. Whole-genome sequencing was used to characterize the genetic modification comprising a sequence from the pUB110 shuttle vector (GenBank: M19465.1), harbouring antimicrobial resistance genes conferring a resistance to kanamycine, neomycin and bleomycin, flanked on each side by a sequence coding for a protease (GenBank: WP_032874795.1). In addition, based on these data, two real-time PCR methods, that can be used by enforcement laboratories, specific to this unauthorized genetically modified bacterium were developed and validated. The present study emphasizes the key role that whole-genome sequencing can take for detection of unknown and unauthorized genetically modified microorganisms in commercialized microbial fermentation products intended for the food and feed chain. Moreover, current issues encountered by the Competent Authorities and enforcement laboratories with such unexpected contaminations and the importance of performing official controls were highlighted.

## Introduction

In the food and feed industry, enzymes, additives and flavourings are frequently produced through fermentation processes involving genetically modified microorganisms (GMM) harbouring antimicrobial resistance (AMR) genes as selection markers^1–10^. Even though viable GMM, or associated recombinant DNA, should be absent in the commercialized microbial fermentation products^11–14^, such accidental contaminations on the European (EU) market have already been reported in 2014, 2018 and 2019 by enforcement laboratories (RASFF 2014.1249, RASFF 2014.1360, RASFF 2014.1657, RASFF 2018.2755, RASFF 2019.0793 and RASFF 2019.3216)^15^. Since no GMM has currently been authorized on the EU market for a commercialization in the food and feed chain, such contaminated microbial fermentation products are therefore automatically considered as containing unauthorized genetically modified organisms (GMO) according to regulation EC/1829/2003 related to commercialization of GMO as food and feed on the EU market^16^. In addition to respecting the EU legislation, the seriousness of this problem is strongly emphasized by public health and environmental concerns related to the presence of AMR genes in the food and feed chain. Indeed, AMR genes can be horizontally transferred to pathogens and gut microbiota. The likelihood of occurrence is especially increased with the presence of the full-length gene, the gene with flanking regions, the gene on mobile genetic elements and the viable GMM harbouring the gene^17–19^. Consequently, the Competent Authorities increasingly consider such accidental GMM contaminations in microbial fermentation products as a critical issue for the safety of the food and feed chain.

Despite its importance, the possibility for enforcement laboratories to perform such control is currently curbed, mainly due to the confidentially of the related GMM dossiers as well as the associated sequences. Moreover, contrarily to commercialized GMO directly intended for the food and feed chain on the EU market, no method specific to the genetically modified (GM) event is here required from developers^16,20–22^. Only few methods targeting such GMM, including two real-time PCR methods specific to the unauthorized vitamin B2-producing GM *B. subtilis* RASFF 2014.1249 strain, are therefore currently available to enforcement laboratories^5,6^.

In this context, the development of a similar approach than for GM plants, consisting in a first-line screening targeting generic transgenic elements to evaluate their potential presence, is therefore needed in order to cover a large spectrum of potential GMM contaminations in microbial fermentation products^22–24^. Therefore, a first-line screening strategy for GMM has recently been proposed. On the one hand, the detection of a potential bacterial contamination with identification at the genus/species level can be performed by Sanger sequencing of the bacterial 16 S rRNA gene region, earlier amplified by PCR^8^. On the other hand, based on patent analyses, the potential presence of key AMR genes commonly harboured by GMM producing fermentation products can be screened by real-time PCR and their full-length size can then be assessed by nested-PCR associated to Sanger sequencing in order to provide information about potential health and environmental risks related to the tested microbial fermentation products^9,10^.

Regarding the second-line analysis to identify these GMM (i.e., GM-event specific and transgenic construct-specific methods), the situation at the legislative and analytical levels is similar to the one encountered with unauthorized GM crops. Therefore, only two real-time PCR methods specific to the unauthorized vitamin B2-producing GM *B. subtilis* RASFF 2014.1249 strain are currently available^5,6^. For all other GMM, further analysis, such as whole-genome sequencing (WGS) or DNA walking coupled to next-generation sequencing (NGS), is needed for their identification^5–7,25–29^.

This issue was recently encountered with a food enzyme (protease) product commercialized on the EU market, in which the unexpected presence of both a full-length AMR gene and a viable bacterial strain was observed^8,10^. No evidence for the presence of GMM could however be established in spite of strong suspicions. To overcome such issue, WGS combined to a *de novo* assembly analysis was applied in this study, allowing therefore to demonstrate a GMM contamination in the food enzyme product. In addition, based on the characterization of the transgenic insertion, event-specific real-time PCR methods were developed and validated in terms of specificity, sensitivity and applicability, to provide to enforcement laboratories a straightforward and rapid detection method for this particular GMM.

## Results and Discussion

**Figure 1:**
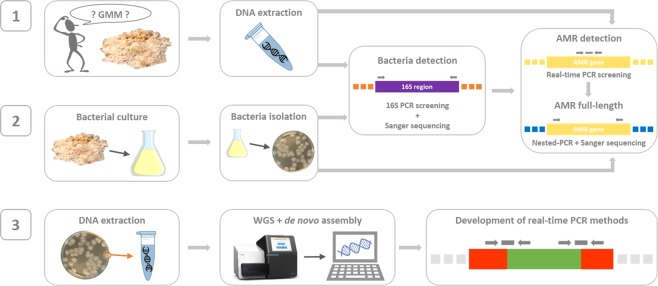
Schematic representation of the workflow, composed of three main successive steps, applied on the tested food enzyme product, allowing to identify unauthorized GMM by whole-genome sequencing (WGS) and subsequently to develop event-specific real-time PCR methods. (1) DNA extracted from the FE preparation was tested for the presence of bacterial DNA as well as the presence of AMR genes frequently harboured by GMM used to produce FE. (2) Living microbial strains, earlier isolated from the FE preparation, were tested for the presence of bacterial DNA and subsequent determination of their genus/species as well as for the presence of AMR genes frequently harboured by GMM used to produce FE. (3) The bacterial strains identified in (2) as carrying AMR genes was characterized by a WGS strategy using a *de novo* assembly analysis in order to demonstrate the presence of a viable unauthorized GMM in the tested FE preparation. With the generated sequences, real-time PCR methods specific to this GMM were developed to be used by enforcement laboratories.

A food enzyme (protease) product commercialized on the EU market, used in this study, was recently suspected to be contaminated by unauthorized GMM. On the one hand, starting from DNA extracted from the food enzyme matrix, the presence of bacterial DNA belonging to the *Bacillus* genus was demonstrated by PCR amplification and Sanger sequencing of the bacterial 16 S rRNA gene region^8^ (Fig. 1 step 1). In addition, the potential GM nature of this contamination was suspected based on a positive real-time PCR signal for the aminoglycoside adenyltransferase (*aadD*) gene conferring a resistance to both kanamycin (KanR) and neomycine (NeoR) (GenBank: M19465.1) (Fig. 1 step 1)^10^. The presence of the full-length size of this *aadD* gene was subsequently confirmed through nested-PCR combined with Sanger sequencing, highlighting potential health and environmental risks associated to this food enzyme product in light of AMR acquisition concerns^9,10^. On the other hand, importantly, a viable bacterial strain was isolated from the tested food enzyme product^8^. This bacterial strain was subjected to the same analysis as above applied on DNA extracted from the food enzyme matrix. Similarly to the results observed using DNA extracted from the food enzyme matrix, this bacterial strain was shown to belong to the Bacillus genus^8^ as well as to carry the *aadD* gene (Fig. 1 step 2, Table 1, Supplementary file 1). The presence of a viable GM Bacillus strain in the tested sample was consequently strongly suspected, but, these results were insufficient to undoubtedly prove the presence of this GMM and characterize its associated genetic modifications.

**Table 1:** Oligonucleotides used for PCR-based methods.

Method	Target	Oligonucleotides					Reference
		Name	Sequences	Concentration	Annealing temperature	Amplicon size	
PCR	Left pUB110 junction of RASFF 2019.3332	Left_junction_long-F	CCCACAATAAATCCCCCTTT	400 nM	60 °C	1 140 bp	This study
		Left_junction_long-R	AAGCCGTCTGTACGTTCCTT	400 nM			
PCR	Right pUB110 junction of RASFF 2019.3332	Right_junction_long-F	TTGGCAAGGGTTTAAAGGTG	400 nM	60 °C	984 bp	This study
		Right_junction_long-R	TTTACGGCTCTCAAGACG	400 nM			
PCR	Left protease junction of RASFF 2019.3332	Left_protease-F	CGAAGTCGGGGGTATTTACA	400 nM	60 °C	535 bp	This study
		Left_protease -R	TCCCGATCGTCTTTTTCAAG	400 nM			
PCR	Right protease junction of RASFF 2019.3332	Right_protease -F	GGGGAAAAATGTTCCGATTT	400 nM	60 °C	491 bp	This study
		Right_protease -R	CAGCAGCTTCCCGTAATACC	400 nM			
Nested-PCR	*cat* gene	cat-F1	TTTGAACCAACAAACGACTTT	400 nM	60 °C	573 bp	^9^
		cat-R1	GGCCTATCTGACAATTCCTGA	400 nM			
		cat-F2	CCAACAAACGACTTTTAGTATAACC	400 nM		529 bp	
		cat-R2	TCCTGCATGATAACCATCAC	400 nM			
Nested-PCR	*aadD* gene	aadD-F1	GAATATTGGATAAATATGGGGATGA	400 nM	60 °C	664 bp	^10^
		aadD-R1	TATCCGTGTCGTTCTGTCCA	400 nM			
		aadD-F2	ATGGCTCTCTTGGTCGTCAG	400 nM		597 bp	
		aadD-R2	CCTGAATCCCATTCCAGAAA	400 nM			
Real-time PCR	*cat* gene	cat-F	GTGACAAGGGTGATAAACTCAAATAC	400 nM	64 °C	96 bp	^9,60^
		cat-P	FAM-ACCTAACTCTCCGTCGCTATTGTAACCAGT-TAMRA	200 nM			
		cat-R	TGTATAAAGTGGCTCTAACTTATCCC	400 nM			
Real-time PCR	*aadD* gene	aadD-F	ATCAGATTGGCCGCTTACAC	400 nM	60 °C	138 bp	^10^
		aadD-P	FAM-CGGTAGAAGCCCAAACGTTCCAC-TAMRA	200 nM			
		aadD-R	ATAAGGGCACAAATCGCATC	400 nM			
Real-time PCR	Left junction of RASFF 2019.3332	Left_junction-F	CGAGAATGCAGCTGAAACAG	400 nM	60 °C	94 bp	This study
		Left_junction-P	FAM-GGACGGACAGATCAAGAACTGTTATGG-TAMRA	200 nM			
		Left_junction-R	CATATGCTCGGGGAATTTATCT	400 nM			
Real-time PCR	Right junction of RASFF 2019.3332	Right_junction-F	GAAAAACGAGGAAAGATGCTG	400 nM	60 °C	115 bp	This study
		Right_junction-P	FAM-GAGCAACTTCAGTTTTCATTTGGAATGG-TAMRA	200 nM			
		Right_junction-R	ACGGTTTTCCGTTTGAAGG	400 nM			

### GMM identification using WGS

Using an Illumina MiSeq system (250 bp paired-end reads), WGS applied on DNA from the bacterial strain isolated from the food enzyme product (Fig. 1 step 3, Supplementary file 2) generated 714,637 paired-end raw reads. Following read trimming, 589,817 high-quality reads (average Phred score of 37) were retained to perform a *de novo* assembly, allowing to generate 430 contiguous sequences (contigs) of which 47 were longer than 1,000 bases with a k-mer coverage of at least 10x. Contig sizes ranged from 56 bp to 457,195 bp, with an N50 value of 291,658.

On the one hand, the generated contigs presented a correspondence to the *Bacillus* genus, and, more precisely, surprisingly to the *B. velezensis* species (RefSeq: NZ_CP011937.1) instead of the expected *B. subtilis* species that was labelled as being the producer organism of the commercialized neutral protease. This identification was based on three observations. Firstly, when using the assembly for typing against the *B. subtilis* MLST schema hosted by the PubMLST.org web-based platform, a perfect match to sequence type 140 was obtained for which only a single isolate was present in the database (PubMLST: ATCC 12321) annotated as the species *B. velezensis*. Secondly, a k-mer based classification of sequencing reads against an in-house dump of all complete genomes in the RefSeq Microbial Genomes database indicated the presence of *B. velezensis* (Supplementary file 3). Thirdly, this identification was confirmed by performing a read mapping analysis to the NCBI representative *B. velezensis* reference genome sequence (RefSeq: NZ_CP001937.1), with a median depth and breadth of coverage of respectively 58x and 94.58% (Supplementary file 4). *B. velezensis* species is not listed by EFSA (2018) as being used in the food and feed industry to produce food and feed additives, enzymes and flavourings intended for the EU market^30^. However, this species, for which the wild-type is harmless for human and closely related to *B. amyloliquefaciens* and *B. subtilis*, has previously been described as highly valuable for producing enzymes, including proteases, for the agro-industrial sector^31–36^.

**Figure 2:**
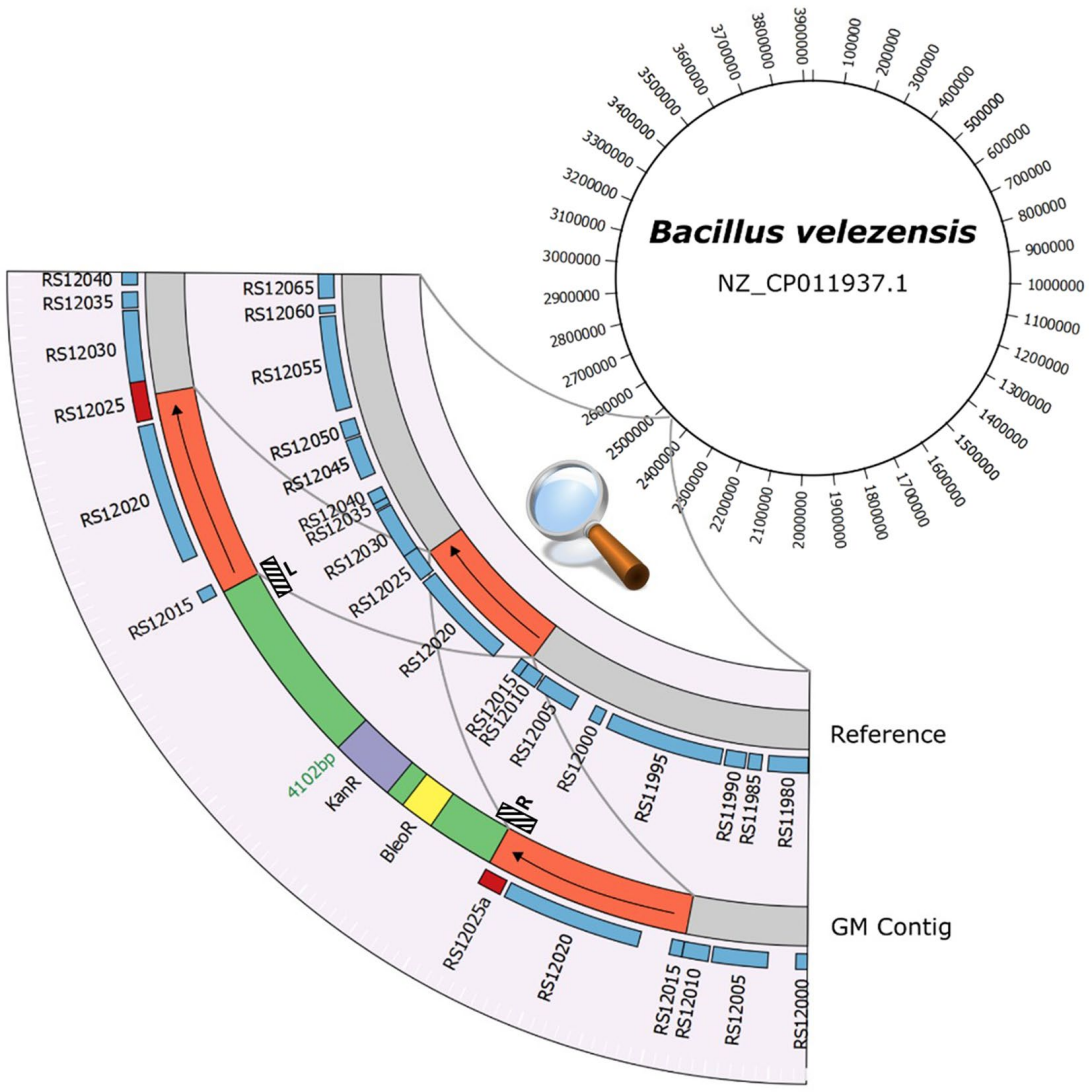
Schematic representation of the identified transgenic insertion. The pUB110 shuttle vector (green) harbours the *aadD* gene conferring a resistance to kanamycin (KanR) (purple) and the *ble* gene conferring a resistance to bleomycin (BleoR) (yellow). Blue rectangles represent annotated genes on the reference genome. The region indicated in orange contains a gene coding for a protease (RS12020) and a part of a gene coding for an acetyltransferase (RS12025). The latter, indicated by a small dark red rectangle in the GM consists out of a full (RS12025) and interrupted (RS12025a) copy. The red region is unique in the wild-type while this red region is duplicated, on both sides of the pUB110 shuttle vector, in the GMM. The dark and hatched rectangles indicate the regions targeted by the left (L) and right (R) event-specific real-time PCR methods developed and validated in this study.

On the other hand, the generated contigs were blasted against the *aadD* gene, conferring KanR and NeoR, that was earlier detected by real-time PCR as well as nested-PCR followed by Sanger sequencing analysis^10^ (Table 1, Supplementary file 1). A contig of 349,285 bp with a k-mer coverage of 59.434x was identified as harbouring the targeted AMR gene (Fig. 2, Supplementary file 5). In order to identify the putative transgenic insertion, the regions flanking this AMR gene were then characterized and compared to the reference genome of *B. velezensis* (RefSeq: NZ_CP011937.1). In the reference genome, a region of 2,385 bp from position 2,460,164 to 2,462,548, with >99% sequence identity, composed of a gene coding for a protease (GenPept: WP_032874795.1; RS12020 in Fig. 2) as well as part of a gene coding for an acetyltransferase (GenPept: WP_032874793.1; RS12025 in Fig. 2), was replaced by a fragment of 9,141 bp in the genome of the isolated bacterial strain containing the region of 2,385 bp in duplicate. Since the tested food enzyme product was commercialized as a protease, the duplication of this region can therefore be explained by the aim of the manufacturers to increase protease yield during the production process. Between these duplicated regions, a sequence of 4,102 bp, with a query coverage and identity of 100%, matching to the pUB110 shuttle vector (GenBank: M19465.1) harbouring the *aadD* gene (GenBank: AAA88361.1), conferring KanR and NeoR, that was earlier identified by real-time PCR and nested-PCR (Fig. 1), and the *ble* gene (RefSeq: NG_047557.1), conferring a resistance to bleomycin (BleoR) was characterized (Fig. 2, Supplementary file 5). This pUB110 shuttle vector, originating from *Staphylococcus aureus*, and the identified AMR genes were previously reported as being highly used in GMM producing bacterial fermentation products in the food and feed chain, especially for selection of strains of interest^10,37^. In addition, the observed left and right transgene flanking regions of the inserted fragment of 9,141 bp as well as the left and right transgene flanking regions of the pUB110 shuttle vector were confirmed by PCR followed by Sanger sequencing (Supplementary files 5,6). Based on all these results, the presence of a genetic modification specific to a viable GMM in the commercialized food enzyme product was therefore demonstrated. These results, communicated to the Belgian Federal Agency for the Safety of the Food Chain, have led to the RASFF 2019.3332 notification at the EU level.

Regarding the bioinformatics methodology, compared to a read-mapping analysis, a *de novo* assembly analysis was the most relevant strategy to identify and characterize an unknown and unauthorized GMM for two reasons. Firstly, no reference sequence is required, representing an advantage e.g. in the present study due to the unavailability of a reference sequence for the identified GMM. This approach is also advantageous when the species identity of the GMM host is difficultly identifiable, as exemplified in this study with the *Bacillus* strain^8^. Secondly, through reconstructing a contig containing the transgenic insertion in the wild-type *B. velezensis* genome, an unnatural association of sequence elements could be inferred, providing strong evidence of the presence of a GMM. Without an available reference sequence for a specific GMM, a read-mapping analysis cannot provide this type of crucial information. Indeed, only the presence of sequences belonging either to the pUB110 shuttle vector or to *B. velezensis* could then have been demonstrated, but no link between the pUB110 shuttle vector and *B. velezensis* could have been established (Supplementary file 4).

### Development of event-specific real-time PCR methods based on WGS data

Based on characterization of the transgenic insertion into *B. velezensis*, two event-specific real-time PCR methods were developed and validated, allowing to specifically target cost- and time-efficiently the unauthorized GMM discovered in the present study (Fig. 1 step 3). These two event-specific methods were designed to cover either the left or the right transgene flanking region of the inserted pUB110 shuttle vector (Fig. 2; Table 1; Supplementary file 5). For each real-time PCR method, an amplicon with the expected size and sequence was obtained (Supplementary file 7). The performance of these real-time PCR methods was then investigated.

First, the specificity of these real-time PCR methods was tested using, as positive control, DNA from the isolated GM *B. velezensis* RASFF 2019.3332 strain as well as, as negative controls, DNA from eighty-five wild-type microbial strains frequently used to produce microbial fermentation products^9,10^, DNA from six different wild-type *B. velezensis* strains, DNA from the vitamin B2-producing GM *B. subtilis* RASFF 2014.1249 strain, DNA from plant (*Zea mays*) and DNA from human. As expected, these event-specific real-time PCR methods presented a positive signal only for the positive control, confirming their specificity (Table 2). Second, the sensitivity of these real-time PCR methods was assessed using DNA from the GM *B. velezensis* RASFF 2019.3332 strain at different estimated full genome copy numbers (6 × 10^6^, 6 × 10^4^, 6 × 10^2^, 60, 12, 6, 1, 0.1 and 0) (Table 3). For both real-time PCR methods, a positive signal was observed at as low as one estimated full genome copy, demonstrating their high sensitivity. Third, the applicability of these real-time PCR methods was tested using DNA from the commercialized food enzyme product in which the GM *B. velezensis* RASFF 2019.3332 strain (sample n°1) was detected as well as a commercialized vitamin B2 feed additive product (RASFF 2014.1249) (sample n°2). As expected, both real-time PCR methods presented a positive signal for the sample n°1 and a negative signal for the sample n°2 (Supplementary file 6). Based on all these results, the two proposed event-specific real-time PCR methods were evaluated as specific, sensitive and applicable, allowing enforcement laboratories to easily target the GM *B. velezensis* RASFF 2019.3332 strain in commercialized microbial fermentation products. If necessary, following to additional optimisation and validation steps, these real-time PCR methods could be combined into a duplex assay.

**Table 2:** List of wild-type microorganisms used for specificity assessment of the real-time PCR methods. The presence and absence of amplification are respectively symbolized by “+” and “-”. For each result, the experiment was carried out in duplicate. DNA from the GM *Bacillus subtilis* (RASFF 2014.1249) strain, ninety-one wild-type microbial strains, plant and animal were used as negative control. DNA from the GM *Bacillus velezensis* (RASFF 2019.332) strain was used as positive control.

Kingdom	Genus	Species	Strain number	Event-specific real-time PCR	
				Left junction	Right junction
Fungi	*Aspergillus*	*acidus*	IHEM 26285	−	−
	*Aspergillus*	*aculeatus*	IHEM 05796	−	−
	*Aspergillus*	*fijiensis*	IHEM 22812	−	−
	*Aspergillus*	*melleus*	IHEM 25956	−	−
	*Aspergillus*	*niger*	IHEM 25485	−	−
	*Aspergillus*	*oryzae*	IHEM 25836	−	−
	*Boletus*	*edulis*	MUCL 043104	−	−
	*Candida*	*cylindracea*	MUCL 041387	−	−
	*Candida*	*rugosa*	IHEM 01894	−	−
	*Chaetomium*	*gracile*	MUCL 053569	−	−
	*Cryphonectria*	*parasitica*	MUCL 007956	−	−
	*Disporotrichum*	*dimorphosporum*	MUCL 019341	−	−
	*Fusarium*	*venenatum*	MUCL 055417	−	−
	*Hansenula*	*polymorpha*	MUCL 027761	−	−
	*Humicola*	*insolens*	MUCL 015010	−	−
	*Kluyveromyces*	*lactis*	IHEM 02051	−	−
	*Leptographium*	*procerum*	MUCL 008094	−	−
	*Mucor*	*javanicus*	IHEM 05212	−	−
	*Penicillium*	*camemberti*	IHEM 06648	−	−
	*Penicillium*	*chrysogenum*	IHEM 03414	−	−
	*Penicillium*	*citrinium*	IHEM 26159	−	−
	*Penicillium*	*decumbens*	IHEM 05935	−	−
	*Penicillium*	*funiculosum*	MUCL 014091	−	−
	*Penicillium*	*multicolor*	CBS 501.73	−	−
	*Penicillium*	*roqueforti*	IHEM 20176	−	−
	*Pichia*	*pastori*	MUCL 027793	−	−
	*Rhizomucor*	*miehei*	IHEM 26897	−	−
	*Rhizopus*	*niveus*	ATCC 200757	−	−
	*Rhizopus*	*oryzae*	IHEM 26078	−	−
	*Saccharomyces*	*cerevisiae*	IHEM 25104	−	−
	*Sporobolomyces*	*singularis*	MUCL 027849	−	−
	*Talaromyces*	*cellulolyticus/pinophilus*	IHEM 16004	−	−
	*Talaromyces*	*emersonii*	DSM 2432	−	−
	*Trametes*	*hirsuta*	MUCL 030869	−	−
	*Trichoderma*	*citrinoviride*	IHEM 25858	−	−
	*Trichoderma*	*longibrachiatum*	IHEM 00935	−	−
	*Trichoderma*	*reesei*	IHEM 05651	−	−
	*Trichoderma*	*viride*	IHEM 04146	−	−
Bacteria	*Arthrobacter*	*ramosus*	LMG 17309	−	−
	*Bacillus*	*amyloliquefaciens*	LMG 9814	−	−
	*Bacillus*	*brevis*	LMG 7123	−	−
	*Bacillus*	*cereus*	ATCC 14579	−	−
	*Bacillus*	*circulans*	LMG 6926T	−	−
	*Bacillus*	*coagulans*	LMG 6326	−	−
	*Bacillus*	*firmus*	LMG 7125	−	−
	*Bacillus*	*flexus*	LMG 11155	−	−
	*Bacillus*	*lentus*	TIAC 101	−	−
	*Bacillus*	*licheniformis*	LMG 6933T	−	−
	*Bacillus*	*megaterium*	LMG 7127	−	−
	*Bacillus*	*pumilus*	DSMZ 1794	−	−
	*Bacillus*	*smithii*	LMG 6327	−	−
	*Bacillus*	*subtilis*	LMG 7135T	−	−
	*Bacillus*	*subtilis*	W04-510	−	−
	*Bacillus*	*subtilis*	E07-505	−	−
	*Bacillus*	*subtilis*	S10005	−	−
	*Bacillus*	*subtilis*	SUB033	−	−
Bacteria	*Bacillus*	*subtilis*	BNB54	−	−
	*Bacillus*	*subtilis*	GMM from RASFF 2014.1249	−	−
	*Bacillus*	*velezensis*	LMG 12384	−	−
	*Bacillus*	*velezensis*	LMG 17599	−	−
	*Bacillus*	*velezensis*	LMG 22478	−	−
	*Bacillus*	*velezensis*	LMG 23203	−	−
	*Bacillus*	*velezensis*	LMG 26770	−	−
	*Bacillus*	*velezensis*	LMG 27586	−	−
	*Bacillus*	*velezensis*	GMM from RASFF 2019.3332	+	+
	*Cellulosimicrobium*	*cellulans*	LMG 16121	−	−
	*Corynebacterium*	*glutamicum*	LMG 3652	−	−
	*Enterococcus*	*faecium*	LMG 9430	−	−
	*Escherichia*	*coli*	LMG2092T	−	−
	*Geobacillus*	*caldoproteolyticus*	DSM 15730	−	−
	*Geobacillus*	*pallidus*	LMG 11159T	−	−
	*Geobacillus*	*stearothermophilus*	LMG 6939T	−	−
	*Klebsiella*	*pneumoniae*	LMG 3113T	−	−
	*Lactobacillus*	*casei*	LMG 6904	−	−
	*Lactobacillus*	*fermentum*	LMG 6902	−	−
	*Lactobacillus*	*plantarum*	LMG 9208	−	−
	*Lactobacillus*	*rhamnosus*	LMG 18030	−	−
	*Lactococcus*	*lactis*	LMG 6890T	−	−
	*Leuconostoc*	*citreum*	LMG 9824	−	−
	*Microbacterium*	*imperiale*	LMG 20190	−	−
	*Paenibacillus*	*alginolyticus*	LMG 18723	−	−
	*Paenibacillus*	*macerans*	LMG 6324	−	−
	*Protaminobacter*	*rubrum*	CBS 574.77	−	−
	*Pseudomonas*	*amyloderamosa*	ATCC-21262	−	−
	*Pseudomonas*	*fluorescens*	LMG1794T	−	−
	*Pullulanibacillus*	*naganoensis*	LMG 12887	−	−
	*Streptomyces*	*aureofaciens*	LMG 5968	−	−
	*Streptomyces*	*mobaraensis*	DSM 40847	−	−
	*Streptomyces*	*murinus*	LMG 10475	−	−
	*Streptomyces*	*netropsis*	LMG 5977	−	−
	*Streptomyces*	*rubiginosus*	LMG20268	−	−
	*Streptomyces*	*violaceoruber*	LMG 7183	−	−
	*Streptoverticillium*	*mobaraense*	CBS 199.75	−	−
Plantae	*Zea*	*mays*	ERM-BF413ak	−	−
Animalia	*Homo*	*sapiens*	/	−	−

**Table 3:** Sensitivity assessment of real-time PCR methods. For each tested DNA concentration from the GM *Bacillus velezensis* RASFF 2019.3332 strain, the corresponding estimated full genome copy number is indicated. The presence and absence of amplification are respectively symbolized by “+” and “−”. For each result at each DNA concentration, the experiment was carried out in quadruplicate. From 25 to 0.0000025 ng, each replicate generated a positive signal. The means of the observed C_q_ are indicated between brackets. From 0.00000025 to 0 ng, each replicate generated a negative signal.

DNA concentration (ng)	Estimated full genome copy number	Real-time PCR methods	
		Left junction	Right junction
25	6,000,000	**+** (C_q_: 14.2)	**+** (C_q_: 13.5)
0.25	60,000	**+** (C_q_: 20.9)	**+** (C_q_: 19.8)
0.0025	600	**+** (C_q_: 28.3)	**+** (C_q_: 27.0)
0.00025	60	**+** (C_q_: 31.1)	**+** (C_q_: 30.0)
0.00005	12	**+** (C_q_: 33.6)	**+** (C_q_: 32.6)
0.000025	6	**+** (C_q_: 34.7)	**+** (C_q_: 33.5)
0.0000025	1	**+** (C_q_: 37.9)	**+** (C_q_: 37.1)
0.00000025	0.1	**−**	**−**
0	0	**−**	**−**

## Conclusion

Following a first-line PCR-based screening analysis targeting generic markers, including 16S rRNA gene region for bacterial presence and key AMR genes frequently harboured by GMM, the potential presence of GMM in a commercialized food enzyme preparation of protease was previously suspected^8,10^. On this basis, a bacterial strain, isolated from this suspicious sample, and its associated genetic modifications were successfully characterized in this study by WGS combined to a *de novo* assembly analysis. The relevance of the proposed analytical workflow on the tested microbial fermentation product was thus demonstrated, including in particular the crucial role of the first-line PCR-based screening step targeting key AMR genes to assess the potential presence of a GMM. WGS applied on the isolated bacterial strain allowed to generate data in order to fully characterize the transgenic insertion, including the transgene flanking regions and unnatural associations of elements, which indubitably proved the presence of a viable GMM. The present study allowed therefore to demonstrate for the first time the presence of an unknown and unauthorized GMM in food enzyme products commercialized on the EU market. Since the identified GMM is viable and carries full-length AMR genes with flanking regions, health risks related to AMR acquisition clearly need to be considered^17–19^. This finding in conjunction with the previous RASFF notification related to the presence of a living GMM in vitamin B2 feed additives^5,6^ strongly emphasises, in particular to the Competent Authorities, the importance of enforcement laboratories to control microbial fermentation products in order to guarantee the safety of the food and feed chain. For this purpose, while using a technology largely mastered by enforcement laboratories, two real-time PCR methods targeting specifically the protease-producing GMM identified in the analysed food enzyme product have been here developed and validated.

This case study emphasizes also concerns at several levels associated to the freedom of choice for consumers, the traceability and the safety of the food and feed chain. Indeed, the requirements of the current EU legislation are insufficient because no tool to monitor the unauthorized presence of GMM is provided to enforcement laboratories and Competent Authorities. As mandatory for GM plants authorized for commercialization on the EU food and feed chain, available identification methods specific to trace GM strains in microbial fermentation products would be helpful^16^. Due to the lack of appropriate tools and the confidentiality of GMM dossiers, enforcement laboratories are thus not able to verify the respect of criteria recommended by EFSA, such as the absence of AMR genes, and consequently the safety of the food and feed chain^17^.

The success of the present study relied on the use of WGS followed by a *de novo* assembly analysis. Although it is still beyond routine activities for most enforcement laboratories, this approach may yet be considered as cost-and time-efficient and state-of-the-art. It requires however a minimum of bioinformatics infrastructure and expertise for data analysis. Therefore, in case of suspicious samples, it could be envisaged that WGS is performed by “sentinel” enforcement laboratories with the necessary expertise in order to characterize sequences of interest, followed by the development of real-time PCR methods that successively can easily be implemented by other peripheral enforcement laboratories^38–40^. In addition, the proposed WGS strategy is only recommendable for isolated bacterial strains. Nonetheless, the isolation step of cultivable bacterial strains represents a bottleneck. Therefore, targeted sequencing approaches, including DNA walking, or metagenomics approaches can be developed for the detection and characterization of GMM when bacterial isolation is not achieved^25–29^. However, specific requirements are necessary for the use of these culture-independent alternatives. For the targeted sequencing approaches, a minimum of prior knowledge is mandatory. On this basis, a DNA walking strategy can be developed to anchor on key AMR genes earlier detected during the first-line PCR-based screening. The unknown regions surrounding these AMR genes can thus be characterized, as previously performed for unknown and unauthorized GM plants^25–29^. For metagenomics, even if no prior knowledge is required similarly to WGS, this promising approach is still currently in its infancy mainly due to the following bottlenecks that are challenging its implementation^26,41–46^. Indeed, the low abundance of DNA of interest present in the total DNA extract, as frequently encountered with GMO contamination, complicated its identification. To overcome such issue, a very high sequencing depth is usually required, increasing dramatically the time and the cost of the analysis. Moreover, metagenomics requires significant development at both wet- and dry-lab levels as well as important computational capacities for such type of complex bioinformatics analysis. However, all these limitations are expected to be overcome in the near future as successfully illustrated by pioneer studies in other problematics, including foodborne pathogen detection^41–46^.

## Materials and methods

### GMM isolation of the tested FE product

The commercialized FE product containing a neutral protease in a maltodextrin and corn starch carrier (Pureferm, Batch number TAP25114738, The Alchemist’s Pantry, https://thealchemistspantry.com/product/pureferm/) in a solid powder form was collected. This FE product (1 g) was mixed into Brain-Heart Infusion broth for bacterial growing overnight at 37 °C. Following a 1:10,000 dilution, 100 µl of this liquid was plated on nutrient agar for bacterial growth overnight at 37 °C.

### Microbial strains

Eighty-five wild-type bacterial and fungal strains were obtained from several collections including Sciensano, the Belgian co-ordinated collections of micro-organisms, the Research Institute for Agriculture, Fisheries and Food, the Convention of Biological Diversity, the American Type Culture Collection and the Deutsche Sammlung von Mikroorganismen und Zellkulturen GmbH (Table 2). These microbial strains correspond to the majority of microorganisms reported by EFSA (2018) as being used in the food and feed industry to produce food and feed additives, enzymes and flavourings^9,30^. Six wild-type *Bacillus velezensis* strains were also collected from the Belgian co-ordinated collections of micro-organisms (Table 2). The vitamin B2-producing GM *B. subtilis* RASFF 2014.1249 strain, previously isolated from a commercialized feed additive, was obtained from Sciensano collection (Table 1). The GM *B. velezensis* isolated in this study was associated to the RASFF 2019.3332 notification number (Table 1).

### DNA extraction, concentration and purity

DNA extraction from wild-type microbial strains (Table 2), the GM *B. subtilis* RASFF 2014.1249 strain (Table 2), the commercialized food enzyme product and the wild-type *Zea mays* was performed as previously described^8–10,47^. Human DNA was purchased from ThermoFisher (4312660) (Table 2).

According to the manufacturer’s instructions, DNA from the bacterial isolate of the GM *B. velezensis* RASFF 2019.3332 strain was extracted using the Genomic-tip 100/G kit (QIAGEN) and then visualized by capillary electrophoresis using the Tapestation 4200 device with the associated genomic DNA Screen Tape and reagents (Agilent) (Supplementary file 2).

Each DNA concentration was measured by spectrophotometry using Nanodrop® 2000 (ThermoFisher) and each DNA purity was evaluated using the A260/A280 and A260/A230 ratios.

### WGS analysis

The DNA library was prepared using the Nextera XT DNA library preparation kit (Illumina) according to manufacturer’s instructions. The sequencing was carried out on an Illumina MiSeq system with the V3 chemistry, obtaining 250 bp paired-end reads. The generated data (SRA number: PRJNA575813) were analyzed via an in-house instance of the Galaxy Workflow Management System^48^, for which a public instance is also available at https://galaxy.sciensano.be. The quality of the generated raw data was evaluated using FastQC 0.11.4 with default parameters. The raw data were trimmed with Trimmomatic 0.36^49^ with SLIDINGWINDOW:4:20 and MINLEN:150 as settings. The quality of the trimmed reads was evaluated using FastQC with default parameters.

For the de novo assembly, contigs were generated from the trimmed reads using SPAdes 3.8^50^ with the starting k-mers set at 117, 121 and 127 (other parameters were left at default values). These k-mer values were selected using VelvetOptimiser 2.5.5^51^ with default settings to optimize for the largest assembly N50. Additional assembly statistics were afterwards generated using Quast 4.1^52^.

For species identification, the assembled contigs were used for typing against the *B. subtilis* MLST schema using the PubMLST.org web-based platform^53^. Additionally, the trimmed paired reads were analyzed with Kraken2 2.0.7-beta^54^ with default parameters against an in-house dump of all complete genomes from the NCBI RefSeq Microbial Genomes database (database retrieved 18/02/2019)^55^. The output was visualized using Krona^56^ with default parameters (Supplementary file 3). Lastly, trimmed reads were mapped to the *B. velezensis* reference genome (RefSeq: NZ_CP001937.1) using BWA-MEM 0.7.17 with default parameters. The generated results were visualized using Tablet 1.19.09.03 (Supplementary file 5). The median depth and breadth of coverage were calculated by extracting per-position depth values with SAMtools depth 1.9^57^ with the ‘-a’ option enabled on the mapped reads, and then extracting the statistics from the resulting tabular file with an in-house script.

For characterization of the transgenic insertion, the assembly was web-based blasted against the aminoglycoside adenyltransferase (*aadD*) gene (GenBank: M19465.1; AAA88361.1) that confers both KanR and NeoR, identifying a single contig carrying the *aadD* gene. This contig was then blasted using the megablast program against the NCBI nucleotide database with default parameters to identify and characterize the transgenic insertion (Fig. 2, Supplementary file 3). The location of the transgenic insertion was then determined by aligning the flanking regions against the single representative reference genome of *B. velezensis* (RefSeq: NZ_CP011937.1) using a local installation of blastn 2.7.1^58^ with default settings. A visualization focusing on the region containing the transgenic insertion was constructed using Circos 0.69-6 based on the reference genome annotation and blastn results^59^ (Fig. 2). Alignment of the transgenic insertion against the NCBI nucleotide database gave a match of length 4,101 bp and sequence identity of >99% with the pUB110 shuttle vector (GenBank: M37273.1). Lastly, trimmed reads were mapped against the shuttle vector sequence and *de novo* assembly and visualized with Tablet as specified above (Supplementary file 5).

### PCR and nested-PCR assays

Each assay was performed in a standard 25 µl reaction volume containing 1X Green DreamTaq PCR Master Mix (ThermoFisher Scientific), 400 nM of each primer (Eurogentec) and 10 ng of DNA from the isolated GM *B. velezensis* RASFF 2019.3332 strain (Table 1; Supplementary file 1). The PCR program consisted of a single cycle of 1 min at 95 °C (initial denaturation) followed by 35 amplification cycles of 30 sec at 95 °C (denaturation), 30 sec at 60 °C (annealing) and 1 min at 72 °C (extension) and finishing by a single cycle of 5 min at 72 °C (final extension). The run was performed on a Swift MaxPro Thermal Cycler (Esco). For each assay, a “No Template Control” (NTC) was included. The final PCR products were visualized by capillary electrophoresis using the Tapestation 4200 device with the associated D1000 or D5000 Screen Tape and reagents (Agilent) (Supplementary file 6). The generated PCR products were purified using USB ExoSAP-IT PCR Product Cleanup (Affymetrix) according to the manufacturer’s instructions, in order to be sequenced on a Genetic Analyzer 3500 using the Big Dye Terminator Kit v3.1 (Applied Biosystems) (Supplementary file 6).

To verify the WGS data related to the characterization of the genetic modification observed in the isolated bacterial strain, primers were designed on the observed left and right transgene flanking regions of the inserted fragment of 9,141 bp as well as on the left and right transgene flanking regions of the pUB110 shuttle vector using the software Primer3 (Table 1; Supplementary file 5).

### Real-time PCR assays

Each real-time PCR assay was performed in a standard 25 µl reaction volume containing 1X TaqMan® PCR Mastermix (Diagenode), 400 nM of each primer (Eurogentec), 200 nM of the probe and 5 µl of DNA (Table 1). The real-time PCR program consisted of a single cycle of DNA polymerase activation for 10 min at 95 °C followed by 45 amplification cycles of 15 sec at 95 °C (denaturing step) and 1 min at 60 °C or 64 °C (annealing-extension step). All runs were performed on an a CFX96 Touch Real-Time PCR Detection System (BioRad). For each assay, a NTC was included. Primers and probes targeting the AMR genes were previously published^9,10,60^.

For the real-time PCR methods targeting either the left or the right transgene flanking region of the insertion identified in the characterized GM *B. velezensis* (RASFF 2019.3332), primers and probes were designed in this study using the software Primer3 (Fig. 2, Table 1; Supplementary file 5). The performance of the latter was assessed at three levels. For the specificity analysis, 10 ng of DNA extracted from the GM *B. subtilis* RASFF 2014.1249 strain, the GM *B. velezensis* RASFF 2019.3332 strain, ninety-one wild-type microbial strains, wild-type *Zea mays* and *Homo sapiens* were tested in duplicate (Table 2). The amplicon generated for the GM *B. velezensis* RASFF 2019.3332 strain was visualized by capillary electrophoresis using the Tapestation 4200 device with the associated D1000 Screen Tape and reagents (Agilent) according to the manufacturer’s instructions (Supplementary file 7), purified using USB ExoSAP-IT PCR Product Cleanup (Affymetrix) according to the manufacturer’s instructions and sequenced on a Genetic Analyzer 3500 using the Big Dye Terminator Kit v3.1 (Applied Biosystems) (Supplementary file 7). For the sensitivity analysis, DNA from the GM *B. velezensis* RASFF 2019.3332 strain at different estimated full genome copy numbers (6 × 10^6^, 6 × 10^4^, 6 × 10^2^, 60, 12, 6, 1, 0.1 and 0) were tested in quadruplicate (Table 3). The calculation of the estimated full genome copy number was based on the NCBI RefSeq reported genome size of *B. velezensis* (NZ_CP011937.1; 3,929,792 bp) and the formula mentioned in Barbau-Piednoir *et al*.^5^. For the applicability analysis, 10 ng of DNA extracted from the commercialized food enzyme product (Pureferm, The Alchemist’s Pantry) (RASFF 2019.3332) (sample n°1) as well as 10 ng of DNA extracted from a vitamin B2 feed additive product (RASFF 2014.1249) (sample n°2) were tested in duplicate.

## Supplementary information

Supplementary information.

## Data Availability

All data generated or analyzed during this study are included in the published article and its supplementary information files or are available from the corresponding author. Regarding the RASFF2019.3332 notification, information related to the availability of the GM bacterial strain and associated recombinant DNA are available from the corresponding author.
