# Supplementary material for: Identification of an unauthorized genetically modified bacteria in food enzyme through whole-genome sequencing
Source: Sci Rep. 2020 Apr 27;10:7094. doi: 10.1038/s41598-020-63987-5 (PMC7184583; doi:10.1038/s41598-020-63987-5)
Supplement: Supplementary file 1 — Supplementary information. [file 41598_2020_63987_MOESM1_ESM.docx]

**Identification of an unauthorized genetically modified bacteria in food enzyme through whole-genome sequencing**

**Marie-Alice Fraiture ^a^** (Marie-Alice.Fraiture@sciensano.be)

**Bert Bogaerts ^a^** (Bert.Bogaerts@sciensano.be)

**Raf Winand ^a^** (raf.winand@sciensano.be)

**Marie Deckers ^a^** (Marie.Deckers@sciensano.be)

**Nina Papazova ^a^** (nina.papazova@sciensano.be)

**Kevin Vanneste ^a^** (kevin.vanneste@sciensano.be)

**Sigrid C. J. De Keersmaecker ^a^** (sigrid.dekeersmaecker@sciensano.be)

**Nancy H. C. Roosens ^a,*^** (nancy.roosens@sciensano.be)

**^a^** Sciensano, Transversal activities in Applied Genomics (TAG), J. Wytsmanstraat 14, 1050 Brussels, Belgium

* Corresponding author: Nancy Roosens (nancy.roosens@sciensano.be), Sciensano, Transversal activities in Applied Genomics (TAG), J. Wytsmanstraat 14, 1050 Brussels, Belgium. Tel: 00 32 (0) 2 642 52 58. Fax: 00 32 (0) 642 52 93

**Supplementary files**

**Supplementary file 1: Analysis of the potential presence of AMR genes in the isolated GM *B. velezensis* RASFF 2019.3332 strain using real-time PCR and nested PCR methods targeting** **a chloramphenicol acetyl-transferase (*cat*) gene (GenBank: NC_002013.1) and the aminoglycoside adenyltransferase (*aadD*) gene (GenBank: M19465.1).** (A) The presence and absence of PCR amplification are respectively symbolized by “+” and “-“. For each PCR result, the experiment was carried out in duplicate. (B) Visualization of the amplicon size and sequence generated by the nested-PCR method targeting the *aadD* gene. The molecular-weight size marker is going from 25 to 1,500 bp. (C) Alignment of the sequence from the amplicon observed in (B) to the *aadD* gene reference sequence (GenBank: M19465.1).

| **A** |
| --- |
| \|  \| **Real-time PCR** \| \| **Nested-PCR** \| \| \| --- \| --- \| --- \| --- \| --- \| \|  \| ***cat* gene** \| ***aadD* gene** \| ***cat* gene** \| ***aadD* gene** \| \| **GM *B. velezensis* RASFF 2019.3332 strain** \| **-** \| **+** \| **-** \| **+** \| |
| **B** |
| 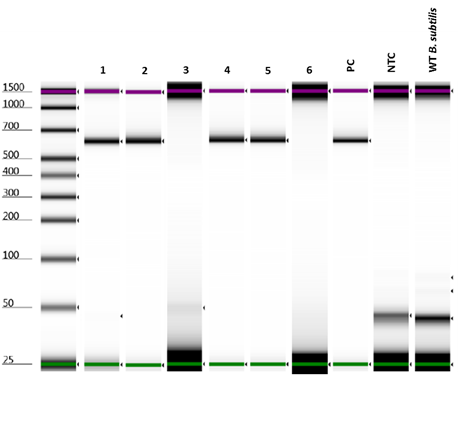 |
| **C** |
| 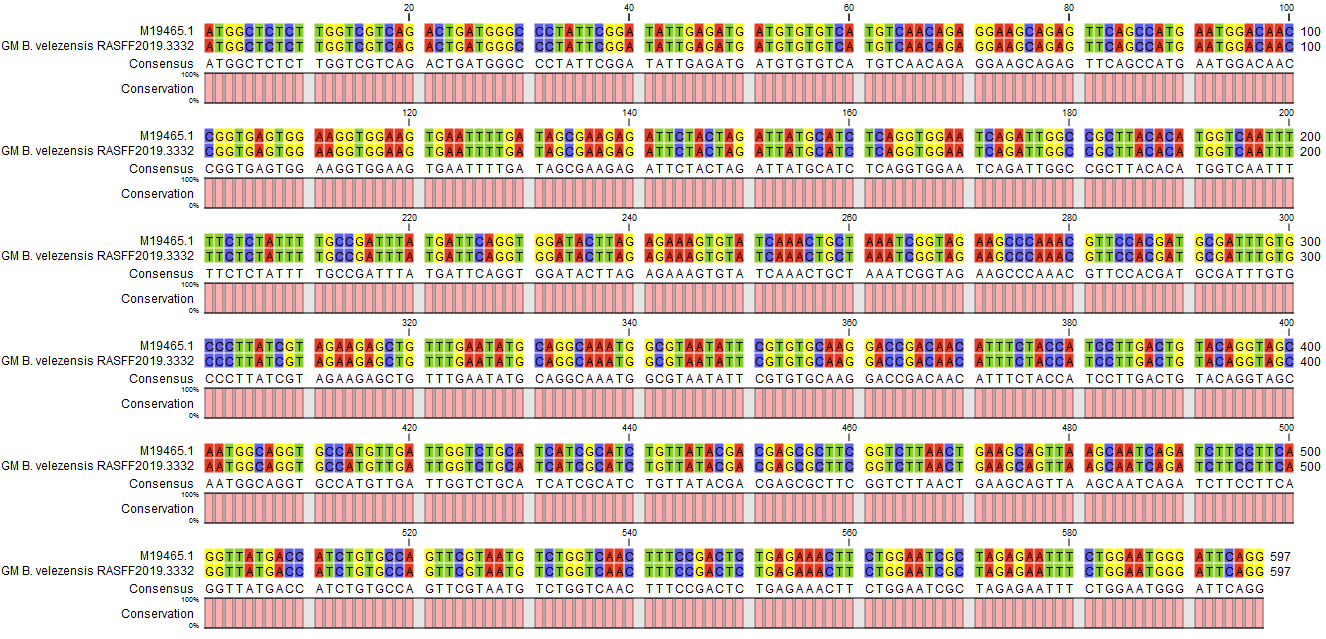 |

**Supplementary file 2: Visualization of DNA extracted from the bacterial isolate of the GM *B. velezensis* RASFF 2019.3332 strain.** The molecular-weight size marker is going from 100 to 48,500 bp. DIN is DNA Integrity Number.

| **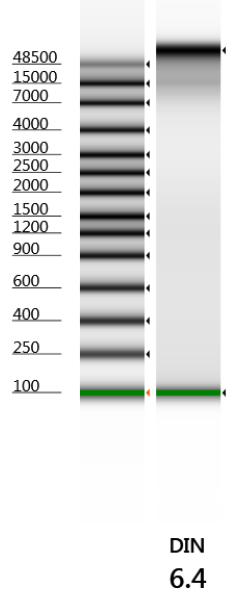** |
| --- |

**Supplementary file 3: Confirmation of the *B. velezensis* identification through k-mer based classification with Kraken2 against an in-house dump of all complete genomes in the RefSeq Microbial Genomes database.** Results were visualized with Krona.

| 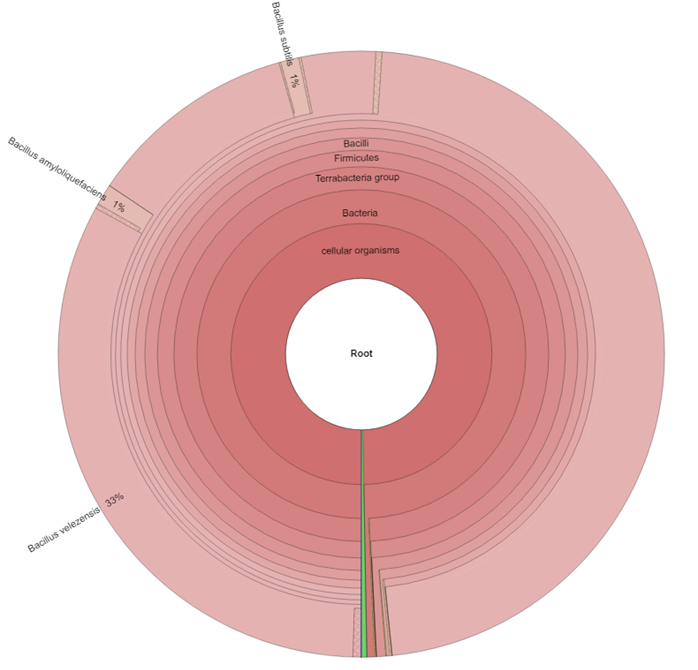 |
| --- |

**Supplementary file 4: Visualization of the read mapping analysis using (A) the reference genome from *B. velezensis* (NZ_CP001937.1), (B) the sequence from the pUB110 shuttle vector (M37273.1) and (C) the contig containing the transgenic insertion.**

| **A** |
| --- |
| **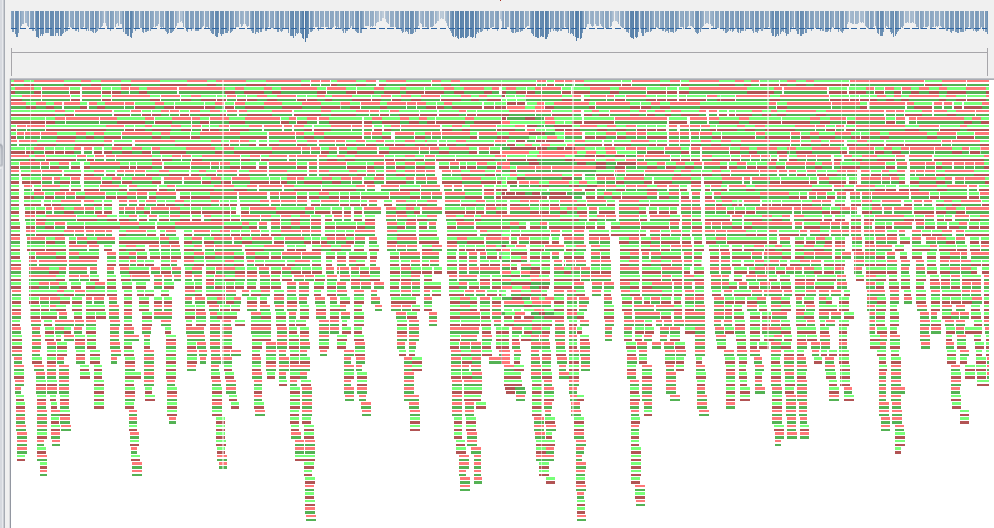** |
| **B** |
| **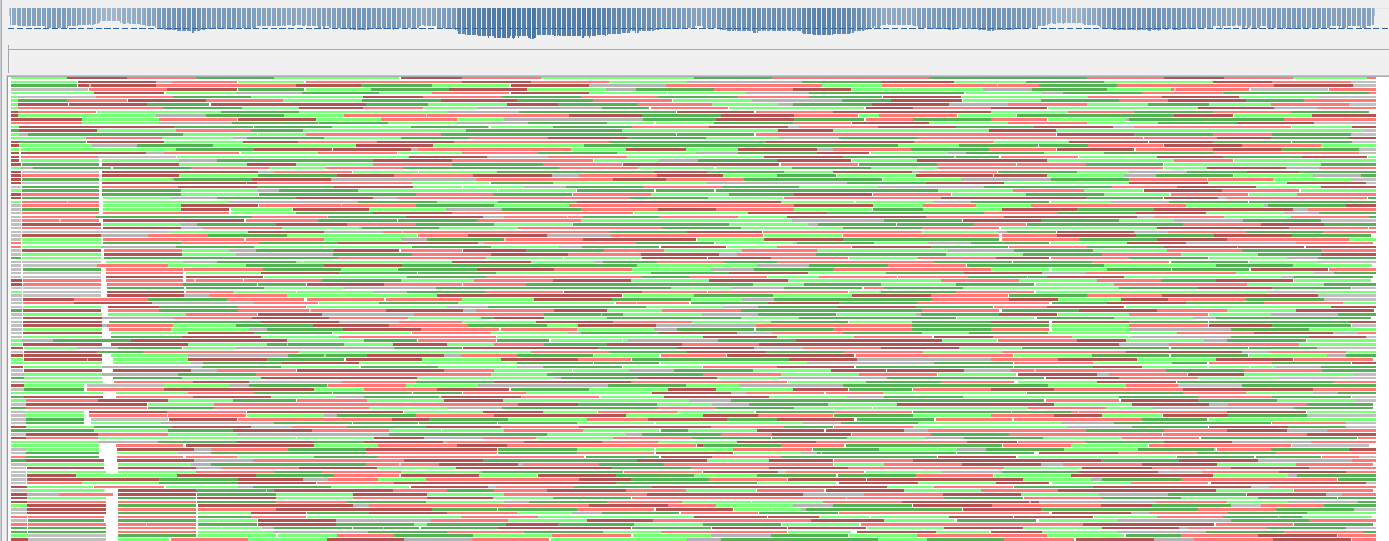** |
| **C** |
| **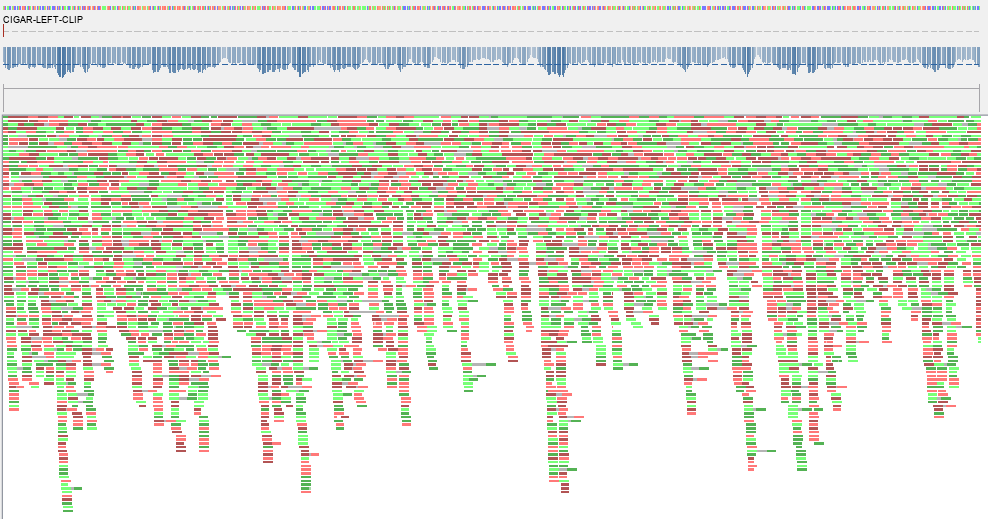** |

**Supplementary file 5: Sequence of the characterized transgenic insertion represented in Figure 2.** The letters in uppercase correspond to the unmodified *Bacillus velezensis* genome while the letters in lowercase correspond to the transgenic insertion. The pUB110 shuttle vector (green) harbours the *aadD* gene conferring KanR (purple) and the *ble* gene conferring BleoR (yellow). The region indicated in red contains a gene coding for a protease (RS12020) and a part of a gene coding for an acetyltransferase (RS12025). The annealing locations of oligonucleotides mentioned in Table 1 are in bold and underlined.

| **A** |
| --- |
| CGGAAAAGGGGCTTATCTGAACAATACGCCGCTTGCTCCATTAAAAGAAGCTTCCATTGAAGAAGCCATTATTGCAATTAATGCCACATGGGTGACGGAAAACAGAAGGATTGACCCGAGTATTTTGGCGCCGCTCGTAAAACGGGTCAGAGGAACGCGTTCCTACGGTTCGGCGGCACTTGAGCTGGCCAGTGTTGCGGCCGGCCGCATTGACGCATACATTACGATGAGGCTCGCGCCGTGGGATTACGCTGCCGGGTGCGTGCTGCTGAA**CGAAGTCGGGGGTATTTACA**CGACCACAGACGGCGAACCGTTTACGTTTTTAGACAACCACAGCGTGCTTGCGGGCAATCCGGCAATACATAAAATTATCTTTGACGACTATTTACATGCTGACAGGTAGAGATTTTGAAGAAGAAGATCTCGGGTTTCTGGAGCAGCTGGCGGCATCACACCCGGTGTGGAAAAGCGAAGAGTTCGGAACAAAAAATGCGGCGGAGTTTATGCTTGCATACTCCATGTACAATGGAACGTGGCTCGTATGGGAGATTGACGGCGCTCCTGCCGCCGtcagttttcatttggaatgggcgccttcaaacggaaaaccgtggctcggaaccgtggccgtcgctccggaggcaaaagagaaaggaatcggacgcgcggtcattgaagagattgcgggccggctgagaggggagcataaagcgatgttttcagccgttcccctggacaggcaggaatggattttgtttttaagccaatgcggctttgaacagcttaagc**ttgaaaaagacgatcggga**aaagacatatatgatcatggtgaagccattgtgaaatgtttcagatataaaaaagagaccggatttctccggtctcttttcttgtttacaagccgaccgcattccaggccgcttctacgcttgcagcgtcttgagagccgtaaaggtcccgcgctgattgaatcaaagctgcttttgcatctttaaagcttgatgacggagtgagatataccgtcagtgcacggtagtaaatctgctccgcttttttcacgccgatttttgtaatcgtgttgtaagcggctttgttcggaattccgctgtttgtatgcacgccgccgtagtcgccggcatcagtattcggaaggtttcgataatttttgtaatggtcgggctgtccgtattttgtcggattggataaactgcggagagccggctggctgaccgtaatatcttcaccgatatcccagtcctcagtatcattgaagtatccgaatacatcagagaaggattcgtttaaagcacccggttgattttcatagttcaggttggctgtttcctgtgtaacaccgtgtgtcatttcatgggccgttacgtccattgaaccggaaagaggcgagaagaatgagccgtcaccgtcaccgtaaatcatttggtcgccgatccaggccgcgttgttgtatttgctgccgtaatgaacggaagatacgattttgccgcctttattgtcgtagctgttgcgtttaaacgtctgatagaaataatcgtacactttgccgagattgtaatgcgcatcaacggcagcgcgctgagaagaagttgtgaactggtttgtagtgcttgatacgagcgtgcccggcaggttatattgtcggttttgcagatcgtacgtaataatttgcgttccggtaggtttagaaagatcacgcattacatatttgccgctttcagaagaaatatttaatgagaccgtttttcctttaagagtcgtacctgttccggttgcagcggcatgctccactttgttttgctttttcaggactttccctgtttccgcatcaacggttacttcccagttagctggttccggttcgatgtagcggatggttacatcataggcgagtcggtatttaccgtcttttgtggccgctgctttcagctcggctttgtttttgtttgcaacgttgccgttagagacggcttcaggtgatttgccgattgctttaaaagcatgatccagcgcttgatttgcagataattttttgctgtttgccgttttggcagaagcatcgttgtttaattctccgttaatcgcatagacattgttggatttatcgacgtgaataatgacttgcgagtctttcacaggcacaccgttaacgacaggcacataacggaagtgcttgtagccgagatcatcggtcgtgtggtcaattagcttcagtctctcagaagggttgcctttgaagactttgccgttttgtttcaagtattgcttgattgctttgtcactgactgaaggcaattcagattgcaccaaagaatgcttcggcacaaagttcgtcaggttttctttaagctgaggattctcagcggcctgaacacccggaagactgatggttaaactcataaaggaagcggcgacagcaacagacaatttcttacctaaa**cccacaataaatcccccttt**ttgaaaataatgaaaacttatattgttataataaaccagcattctctcattctgcaatataaaactagactccgctgatgaattgatagaatcgtcccggaaaggcttccggcggccagcatcggggtataagccgagaggcacgtgttgaatcagacatgagtcgaatgatgcgatttattctgaaaaaaatgacaaatgaagtcgggaaaaatgataggggaaaaatgttccgatttgtaaaactttttttatagccgtttgtctaaacgggtgaggccttaataaacgaaaaggattgtttcagatgtcagagattttcagtcattttattcagcatttcgacagatatttcaagagctgtcttacgaagagattgctgatgttttggactggagtctttcgaaggtgaagagcaccctgtacaggg**cgagaatgcagctgaaacag**cacttggcggaaaaaaggaa**ggacggacagatcaagaactgttatgg**ctaca**agataaattccccgagcatatg**aaaaaacagggttttgagttgaagcgtggtgaacgtggctctgaccgtaaacatattgagacagctaaatttaaaaaacaaactttggaaaaagagattgattttctagaaaaaaatttagcagttaaaaaagatgaatggactgcttatagcgataaagttaaatcagatttagaagtaccagcgaaacgacacatgaaaagtgttgaagtgccaacgggtgaaaagtccatgtttggtttgggaaaagaaataatgaaaacagaaaagaaaccaaccaaaaatgttgttatatcggagcgtgattataaaaacttagtgactgctgcgagagataacgataggttaaaacagcatgttagaaatctcatgagtactgatatggcgagagaatataaaaaattaagtaaagaacatgggcaagttaaagaaaaatatagtggtcttgtagagcgatttaatgaaaatgtaaatgattataatgagttgcttgaagaaaacaagtctttaaagtctaaaataagcgatttaaagcgtgatgtgagtttaatctatgaaagcactaaggaattcctt**aaggaacgtacagacggctt**aaaagcctttaaaaacgtttttaaggggtttgtagacaaggtaaaggataaaacagcacaattccaagaaaaacacgatttagaacctaaaaagaacgaatttgaactaactcataaccgagaggtaaaaaaagaacgaagtcgagatcagggaatgagtttataaaataaaaaaagcacctgaaaaggtgtctttttttgatggttttgaacttgttctttcttatcttgatacatatagaaataacgtcatttttattttagttgctgaaaggtgcgttgaagtgttggtatgtatgtgttttaaagtattgaaaacccttaaaattggttgcacagaaaaaccccatctgttaaagttataagtgactaaacaaataactaaatagatgggggtttcttttaatattatgtgtcctaatagtagcatttattcagatgaaaaatcaagggttttagtggacaagacaaaaagtggaaaagtgagaccatggagagaaaagaaaatcgctaatgttgattactttgaacttctgcatattcttgaatttaaaaaggctgaaagagtaaaagattgtgctgaaatattagagtataaacaaaatcgtgaaacaggcgaaagaaagttgtatcgagtgtggttttgtaaatccaggctttgtccaatgtgcaactggaggagagcaatgaaacatggcattcagtcacaaaaggttgttgctgaagttattaaacaaaagccaacagttcgttggttgtttctcacattaacagttaaaaatgtttatgatggcgaagaattaaataagagtttgtcagatatggctcaaggatttcgccgaatgatgcaatataaaaaaattaataaaaatcttgttggttttatgcgtgcaacggaagtgacaataaataataaagataattcttataatcagcacatgcatgtattggtatgtgtggaaccaacttattttaagaatacagaaaactacgtgaatcaaaaacaatggattcaattttggaaaaaggcaatgaaattagactatgatccaaatgtaaaagttcaaatgattcgaccgaaaaataaatataaatcggatatacaatcggcaattgacgaaactgcaaaatatcctgtaaaggatacggattttatgaccgatgatgaagaaaagaatttgaaacgtttgtctgatttggaggaaggtttacaccgtaaaaggttaatctcctatggtggtttgttaaaagaaatacataaaaaattaaaccttgatgacacagaagaaggcgatttgattcatacagatgatgacgaaaaagccgatgaagatggattttctattattgcaatgtggaattgggaacggaaaaattattttattaaagagtagttcaacaaacgggccagtttgttgaagattagatgctataattgttattaaaaggattgaaggatgcttaggaagacgagttattaatagctgaataagaacggtgctctccaaatattcttatttagaaaagcaaatctaaaattatctgaaaagggaatgagaatagtgaatggaccaataataatgactagagaagaaagaatgaagattgttcatgaaattaaggaac**gaatattggataaatatggggatga**tgttaaggctattggtgttt**atggctctcttggtcgtcag**actgatgggccctattcggatattgagatgatgtgtgtcatgtcaacagaggaagcagagttcagccatgaatggacaaccggtgagtggaaggtggaagtgaattttgatagcgaagagattctactagattatgcatctcaggtgga**atcagattggccgcttacac**atggtcaatttttctctattttgccgatttatgattcaggtggatacttagagaaagtgtatcaaactgctaaat**cggtagaagcccaaacgttccacgatgcgatttgtgcccttat**cgtagaagagctgtttgaatatgcaggcaaatggcgtaatattcgtgtgcaaggaccgacaacatttctaccatccttgactgtacaggtagcaatggcaggtgccatgttgattggtctgcatcatcgcatctgttatacgacgagcgcttcggtcttaactgaagcagttaagcaatcagatcttccttcaggttatgaccatctgtgccagttcgtaatgtctggtcaactttccgactctgagaaacttctggaatcgctagagaat**ttctggaatgggattcagg**ag**tggacagaacgacacggata**tatagtggatgtgtcaaaacgcataccattttgaacgatgacctctaataattgttaatcatgttggttacgtatttattaacttctcctagtattagtaattatcatggctgtcatggcgcattaacggaataaagggtgtgcttaaatcgggccattttgcgtaataagaaaaaggattaattatgagcgaattgaattaataataaggtaatagatttacattagaaaatgaaaggggattttatgcgtgagaatgttacagtctatcccggcattgccagtcggggatattaaaaagagtataggtttttattgcgataaactaggtttcactttggttcaccatgaagatggattcgcagttctaatgtgtaatgaggttcggattcatctatgggaggcaagtgatgaaggctggcgctctcgtagtaatgattcaccggtttgtacaggtgcggagtcgtttattgctggtactgctagttgccgcattgaagtagagggaattgatgaattatatcaacatattaagcctttgggcattttgcaccccaatacatcattaaaagatcagtggtgggatgaacgagactttgcagtaattgatcccgacaacaatttgattagcttttttcaacaaataaaaagctaaaatctattattaatctgttcagcaatcgggcgcgattgctgaataaaagatacgagagacctctcttgtatcttttttattttgagtggttttgtccgttacactagaaaaccgaaagacaataaaaattttattcttgctgagtctggctttcggtaagctagacaaaacggacaaaataaaaa**ttggcaagggtttaaaggtg**gagattttttgagtgatcttctcaaaaaatactacctgtcccttgctgatttttaaacgagcacgagagcaaaacccccctttgctgaggtggcagagggcaggtttttttgtttcttttttctcgtaaaaaaaagaaaggtcttaaaggttttatggttttggtcggcactgccgacagcctcgcagagcacacactttatgaatataaagtatagtgtgttatactttacttggaagtggttgccggaaagagcgaaaatgcctcacatttgtgccacctaaaaaggagcgatttacatatgagttatgcagtttgtagaatgcaaaaagtgaaatcagctggactaaaaggcatgcaatttcataatcaaagagagcgaaaaagtagaacgaatgatgatattgaccatgagcgaacacgtgaaaattatgatttgaaaaatgataaaaatattgattacaacgaacgtgtcaaagaaattattgaatcacaaaaaacaggtacaa**gaaaaacgaggaaagatgctg**ttcttgtaaatgagttgctagtaacatctgaccgagattttttt**gagcaacttcagttttcatttggaatgg**gcg**ccttcaaacggaaaaccgt**ggctcggaaccgtggccgtcgctccggaggcaaaagagaaaggaatcggacgcgcggtcattgaagagattgcgggccggctgagaggggagcataaagcgatgttttcagccgttcccctggacaggcaggaatggattttgtttttaagccaatgcggctttgaacagcttaagcttgaaaaagacgatcgggaaaagacatatatgatcatggtgaagccattgtgaaatgtttcagatataaaaaagagaccggatttctccggtctcttttcttgtttacaagccgaccgcattccaggccgcttctacgcttgcag**cgtcttgagagccgtaaa**ggtcccgcgctgattgaatcaaagctgcttttgcatctttaaagcttgatgacggagtgagatataccgtcagtgcacggtagtaaatctgctccgcttttttcacgccgatttttgtaatcgtgttgtaagcggctttgttcggaattccgctgtttgtatgcacgccgccgtagtcgccggcatcagtattcggaaggtttcgataatttttgtaatggtcgggctgtccgtattttgtcggattggataaactgcggagagccggctggctgaccgtaatatcttcaccgatatcccagtcctcagtatcattgaagtatccgaatacatcagagaaggattcgtttaaagcacccggttgattttcatagttcaggttggctgtttcctgtgtaacaccgtgtgtcatttcatgggccgttacgtccattgaaccggaaagaggcgagaagaatgagccgtcaccgtcaccgtaaatcatttggtcgccgatccaggccgcgttgttgtatttgctgccgtaatgaacggaagatacgattttgccgcctttattgtcgtagctgttgcgtttaaacgtctgatagaaataatcgtacactttgccgagattgtaatgcgcatcaacggcagcgcgctgagaagaagttgtgaactggtttgtagtgcttgatacgagcgtgcccggcaggttatattgtcggttttgcagatcgtacgtaataatttgcgttccggtaggtttagaaagatcacgcattacatatttgccgctttcagaagaaatatttaatgagaccgtttttcctttaagagtcgtacctgttccggttgcagcggcatgctccactttgttttgctttttcaggactttccctgtttccgcatcaacggttacttcccagttagctggttccggttcgatgtagcggatggttacatcataggcgagtcggtatttaccgtcttttgtggccgctgctttcagctcggctttgtttttgtttgcaacgttgccgttagagacggcttcaggtgatttgccgattgctttaaaagcatgatccagcgcttgatttgcagataattttttgctgtttgccgttttggcagaagcatcgttgtttaattctccgttaatcgcatagacattgttggatttatcgacgtgaataatgacttgcgagtctttcacaggcacaccgttaacgacaggcacataacggaagtgcttgtagccgagatcatcggtcgtgtggtcaattagcttcagtctctcagaagggttgcctttgaagactttgccgttttgtttcaagtattgcttgattgctttgtcactgactgaaggcaattcagattgcaccaaagaatgcttcggcacaaagttcgtcaggttttctttaagctgaggattctcagcggcctgaacacccggaagactgatggttaaactcataaaggaagcggcgacagcaacagacaatttcttacctaaacccacaataaatccccctttttgaaaataatgaaaacttatattgttataataaaccagcattctctcattctgcaatataaaactagactccgctgatgaattgatagaatcgtcccggaaaggcttccggcggccagcatcggggtataagccgagaggcacgtgttgaatcagacatgagtcgaatgatgcgatttattctgaaaaaaatgacaaatgaagtcgggaaaaatgata**ggggaaaaatgttccgattt**gtaaaactttttttatagccgtttgtctaaacgggtgaggccttaataaacgaaaaggattgtttcagatgtcagagattttcagtcattttattcagcatttcgacagatatttcaagagctgtcttacgaagagattgctgatgttttggactggagtctttcgaaggtgaagagcaccctgtacagggcgagaatgcagctgaaacagcacttggcggaaaaaaggaaggacggacagaTATGACTTGCTATTTAGTGAGAGATTTACTCCCTTTATATATTGAAGGCGATTGTGAAACAGAAACGGAGCGGTTTATTTCCCGCCATCTTGAATCATGCGGTAAGTGCGGGAATTTATATCATATGATGAAGGAGCCGCTTGACCTCGGCAGCCCTGAGATGAAGGCGCCAGCCTGTTATGCAGAAGAAGAAAGGCGGTTTAAGGAAC**GGTATTACGGGAAGCTGCTG**ATAAAAGCAGCCTGTTTGTTCGGCGCTGTTTTCTTTATCATGCTGATCCTTAAACTGCTGATTTAAAAAAACGCCCGGCTGAATGACCGGGCGTTTTAATCCGTTTACTCAGCCGGATGAAAGGCTGTATAAATGCCTTCAAGCAATTCCTGGGGCAGTGAATTAAAGAATCCGGCACACAGCTTTGCTTCTTTTTTTCCTGCTCCTGCTTTTTCAGCAAGGTCCTGATCAAACGTATACACACCGTCTTCTTCAATCAGCGCAGATTGAAGGATATGAAGACTTTGGGAAAAAGCGGTGAACAGCTGATCAACGATAAGGGCCTCTTTTTCCGTAAAGCCGAGCTCCACCGCTTTTTCTTTATTAAATGTATATTCGCCGTCCGTTTTCTCAATACATTGGGAAAGAACGGTTAAAAATTCGGTTGAACTGTCCCACAGCTTTTGAATGA |

**Supplementary file 6: Confirmation of the identified insertion characterized by WGS analysis.** (A) Visualization of the amplicons generated by the PCR methods targeting the left and right protease junctions as well as the left and right pUB110 junctions of the GM *B. velezensis* RASFF 2019.3332 strain (Table 1, Supplementary file 5). The molecular-weight size marker is going from 15 to 10,000 bp or from 25 to 1,500 bp. (B) Alignments of the amplicons observed in (A) against the contig presenting the transgenic insertion obtained by WGS analysis indicated in Supplementary file 5.

| **A** |
| --- |
| **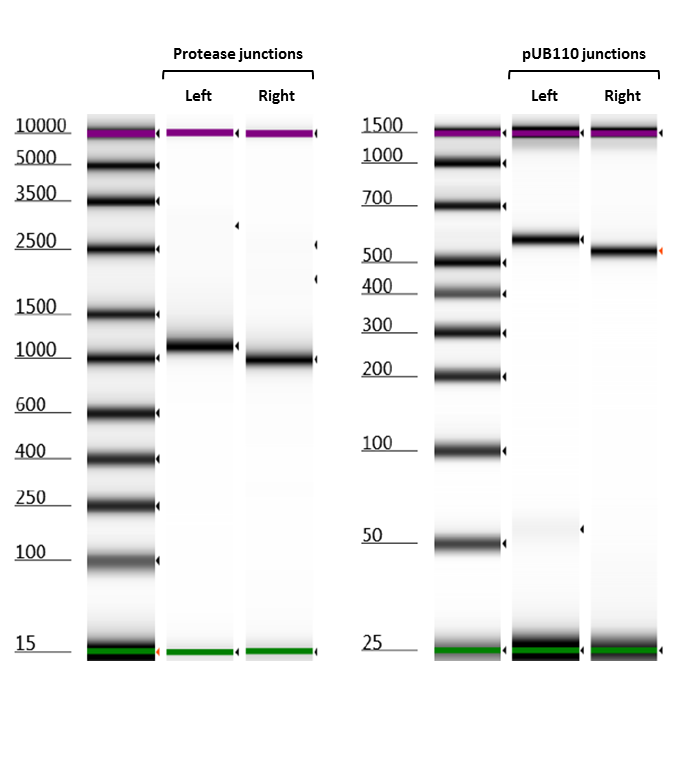** |
| **B** |
| **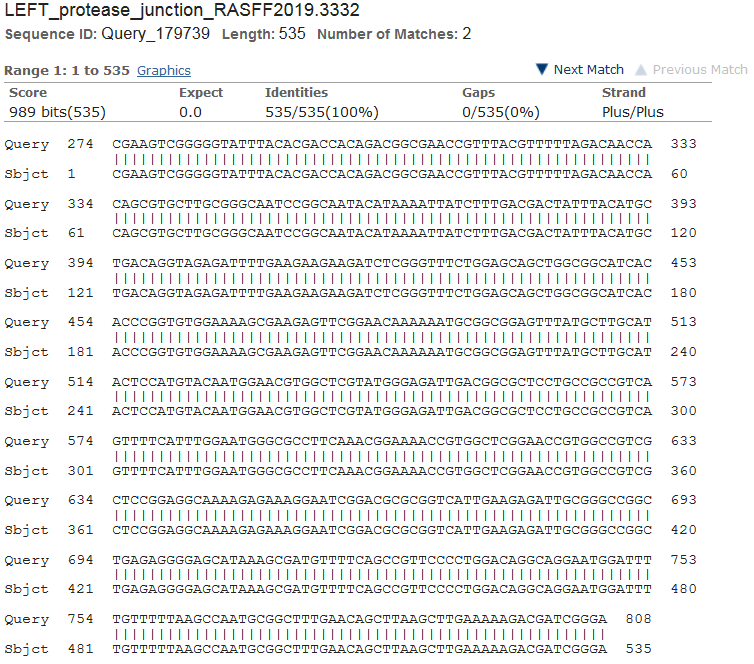** |
| **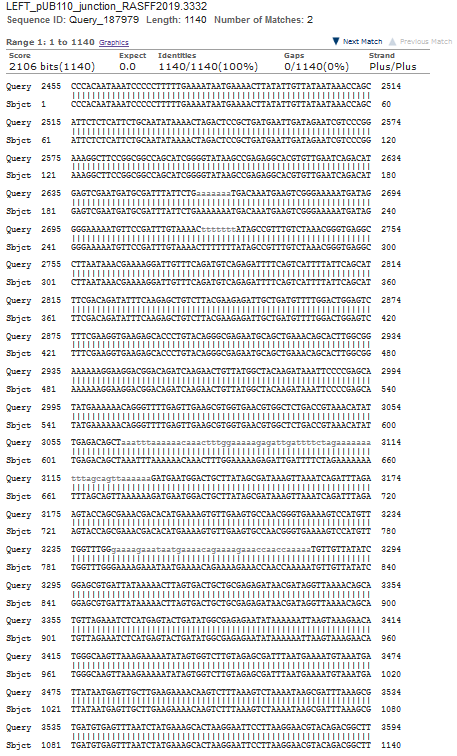** |
| **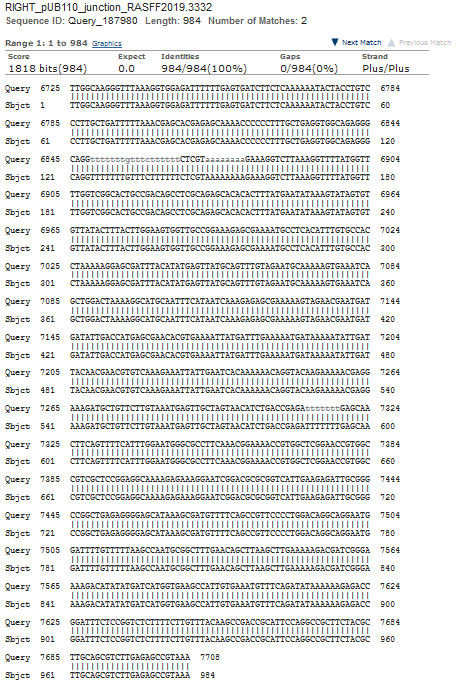** |
| **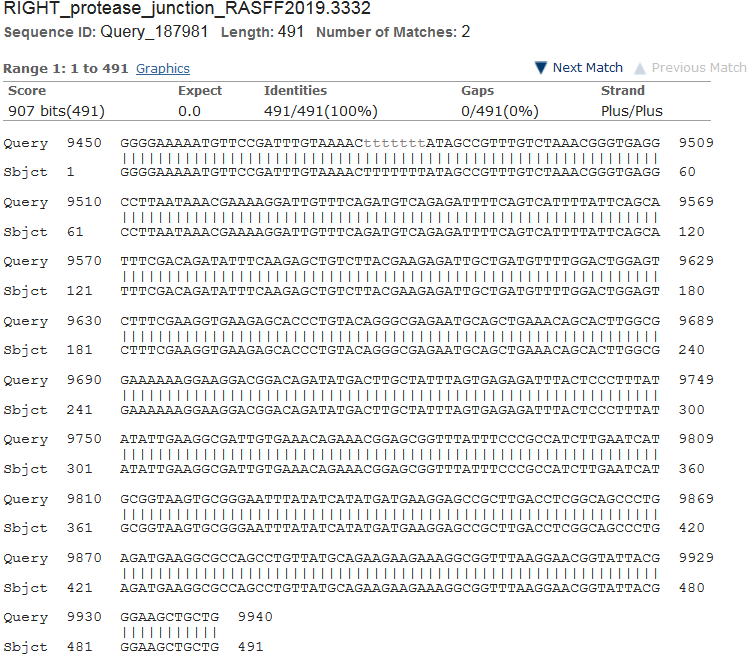** |

**Supplementary file 7: Identity verification of the amplicons generated by the event-specific real-time markers applied on the GM *B. velezensis* RASFF 2019.3332 strain.** (A) Visualization of the amplicons generated by the event-specific real-time PCR method targeting the left or right transgene flanking regions of the identified insertion. The molecular-weight size marker is going from 25 to 1,500 bp. (B) Alignments of the amplicon generated by the event-specific real-time PCR method targeting the left or right transgene flanking regions of the identified insertion against the contig presenting the transgenic insertion obtained by WGS analysis from Supplementary file 5.

| **A** |
| --- |
| 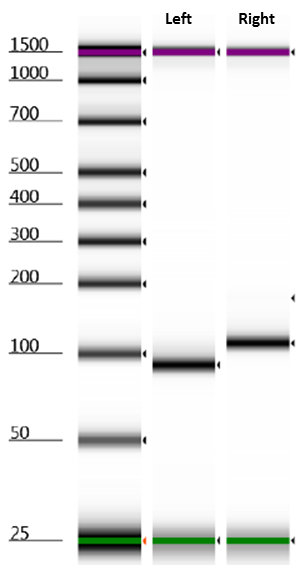 |
| **B** |
| 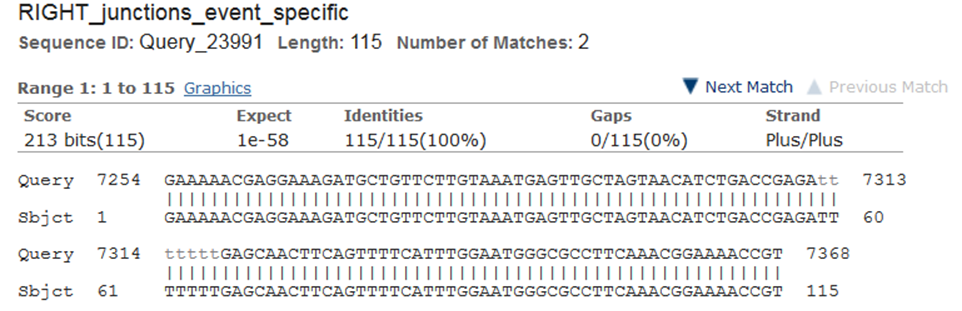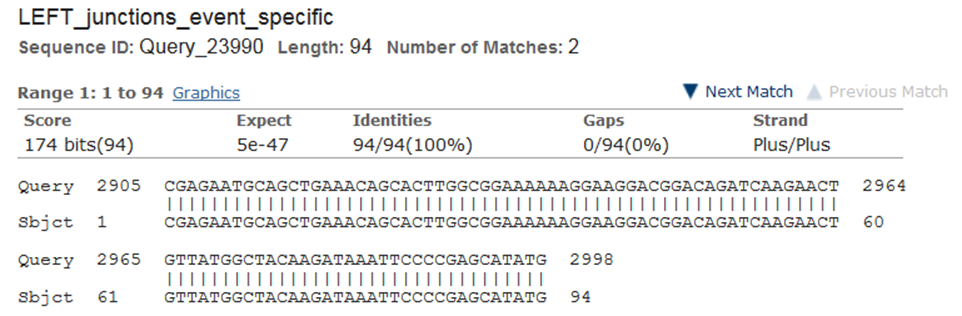 |

**Supplementary file 8: Applicability assessment of real-time PCR methods using commercialized bacterial fermentation products.** Each sample was tested at 10 ng in duplicate. The presence or absence of PCR amplification is respectively symbolized by “+” or “-“. The mean of the observed C_q_ are indicated between brackets. The sample n°2 was used as negative control.

| **Sample n°** | **Sample descriptions** | **Real-time PCR methods** | |
| --- | --- | --- | --- |
|  |  | **Left junction** | **Right junction** |
| **1** | Food enzyme - alpha-amylase (RASFF 2019.3332) | **+**  (C_q_: 12.7) | **+**  (C_q_: 12.0) |
| **2** | Feed additive - vitamin B2 (RASFF 2014.1249) | **-** | **-** |
